# Complete genome sequence of *Nitrosomonas sp.* Is79, an ammonia oxidizing bacterium adapted to low ammonium concentrations

**DOI:** 10.4056/sigs.3517166

**Published:** 2013-02-25

**Authors:** Annette Bollmann, Christopher J. Sedlacek, Jeanette Norton, Hendrikus J. Laanbroek, Yuichi Suwa, Lisa Y. Stein, Martin G. Klotz, Daniel Arp, Luis Sayavedra-Soto, Megan Lu, David Bruce, Chris Detter, Roxanne Tapia, James Han, Tanja Woyke, Susan M. Lucas, Sam Pitluck, Len Pennacchio, Matt Nolan, Miriam L. Land, Marcel Huntemann, Shweta Deshpande, Cliff Han, Amy Chen, Nikos Kyrpides, Konstantinos Mavromatis, Victor Markowitz, Ernest Szeto, Natalia Ivanova, Natalia Mikhailova, Ioanna Pagani, Amrita Pati, Lin Peters, Galina Ovchinnikova, Lynne A. Goodwin

**Affiliations:** 1 Miami University, Oxford, Ohio, USA; 2 Utah State University, Logan, Utah, USA; 3 Netherlands Institute for Ecology, Wageningen, The Netherlands; 4 Chuo University, Tokyo, Japan; 5 University of Alberta, Edmonton, Alberta, Canada; 6 University of North Carolina, Charlotte, North Carolina, USA; 7 Oregon State University, Corvallis, Oregon, USA; 8 Los Alamos National Laboratory, Bioscience Division, Los Alamos, New Mexico, USA; 9 DOE Joint Genome Institute, Walnut Creek, California, USA; 10 Oak Ridge National Laboratory, Oak Ridge, Tennessee, USA

**Keywords:** *Nitrosomonas*, Ammonia-oxidizing bacteria, Ammonia oxidation, nitrification, nitrogen cycle, freshwater, oligotrophic

## Abstract

*Nitrosomonas sp.* Is79 is a chemolithoautotrophic ammonia-oxidizing bacterium that belongs to the family *Nitrosomonadaceae* within the phylum *Proteobacteria*. Ammonia oxidation is the first step of nitrification, an important process in the global nitrogen cycle ultimately resulting in the production of nitrate. *Nitrosomonas sp.* Is79 is an ammonia oxidizer of high interest because it is adapted to low ammonium and can be found in freshwater environments around the world. The 3,783,444-bp chromosome with a total of 3,553 protein coding genes and 44 RNA genes was sequenced by the DOE-Joint Genome Institute Program CSP 2006.

## Introduction

*Nitrosomonas sp.* Is79 is a betaproteobacterial ammonia-oxidizer. The genus name *Nitrosomonas* derived from nitrosus (Latin: nitrous) and monad (Greek: a unit) meaning nitrite producing unit. *Nitrosomonas sp.* Is79 was enriched and isolated from freshwater sediment [1]. Closely related strains can be found in freshwater environments around the world [2-6]. Other *Nitrosomonas* species have been isolated from freshwater and marine systems, wastewater treatments plants and soils [7,8]. The genome sequence of *Nitrosomonas sp.* Is79 is the fifth genome of the betaproteobacterial ammonia oxidizers that has been completed by DOE-Joint Genome Institute (CP002876.1) [9-12]. Here we present summary classification and a set of features for *Nitrosomonas sp.* Is79, together with the description of the complete genome sequence and annotation.

## Classification and features

Fourteen species with valid published names are currently assigned to the *Nitrosomonadaceae* [13-19]. Besides these described species, many undescribed isolates are available [7,20-22]. These strains were isolated from freshwater, marine systems, wastewater and soils, share the traits of aerobic chemolithoautotrophic metabolism using ammonia as an electron donor, and carbon dioxide as carbon source (Table 1).

**Table 1:** Classification and general features of *Nitrosomonas sp.* Is79 according to the MIGS recommendations [23]

**MIGS ID**	**Property**	**Term**	**Evidence code**
	Current	Domain *Bacteria*	TAS [24]
	classification	Phylum *Proteobacteria*	TAS [25]
		Class *Betaproteobacteria*	TAS [26,27]
		Order *Nitrosomonadales*	TAS [27,28]
		Family *Nitrosomonadaceae*	TAS [27,29]
		Genus *Nitrosomonas*	TAS [13,30-32]
		Species *Nitrosomonas* sp	IDA
		Type strain Is79	IDA
	Gram stain	negative	NAS
	Cell shape	rod-shaped, short	NAS
	Motility	not reported	
	Sporulation	none	NAS
	Temperature range	mesophile	NAS
	Optimum temperature	not reported	
	Salinity	< 50mM NaCl, very sensitive to salt	TAS [1,33]
MIGS-22	Oxygen requirement	aerobic	TAS [1]
	Carbon source	carbon dioxide	TAS [1]
	Energy source	ammonia	TAS [1]
	Energy metabolism	chemolithoautotroph	TAS [1]
MIGS-23	Isolation and growth conditions	Isolation after enrichment in chemostat under low substrate concentrations, adapted to low ammonium concentrations in the medium	TAS [1]
MIGS-6	Habitat	freshwater	TAS [1]
MIGS-15	Biotic relationship	free-living	NAS
MIGS-14	Pathogenicity	None	NAS
	Biosafety level	1	TAS [34]
	Isolation	freshwater sediment	TAS [1]
MIGS-4	Geographic location	Lake Drontermeer (Netherlands)	TAS [1]
MIGS-4.1	Latitude	52°58’N	NAS
MIGS-4.2	Longitude	5°50’E	NAS
MIGS-4.3	Depth	0.5 m (root zone in the littoral zone of the lake)	TAS [1]
MIGS-4.4	Altitude	around sea level	TAS [1]
MIGS-5	Sample collection time	Fall 1997	TAS [1]

Evidence codes – IDA: Inferred from Direct Assay (first time in publication); TAS: Traceable Author Statement (i.e. a direct report exists in the literature); NAS: Non-traceable Author Statement (i.e. not directly observed for living, isolated sample, but based on a generally accepted property for the species, or anecdotal evidence). These evidence codes are from a living isolate by one of the authors or an expert mentioned in the acknowledgements.

**Figure 1:**
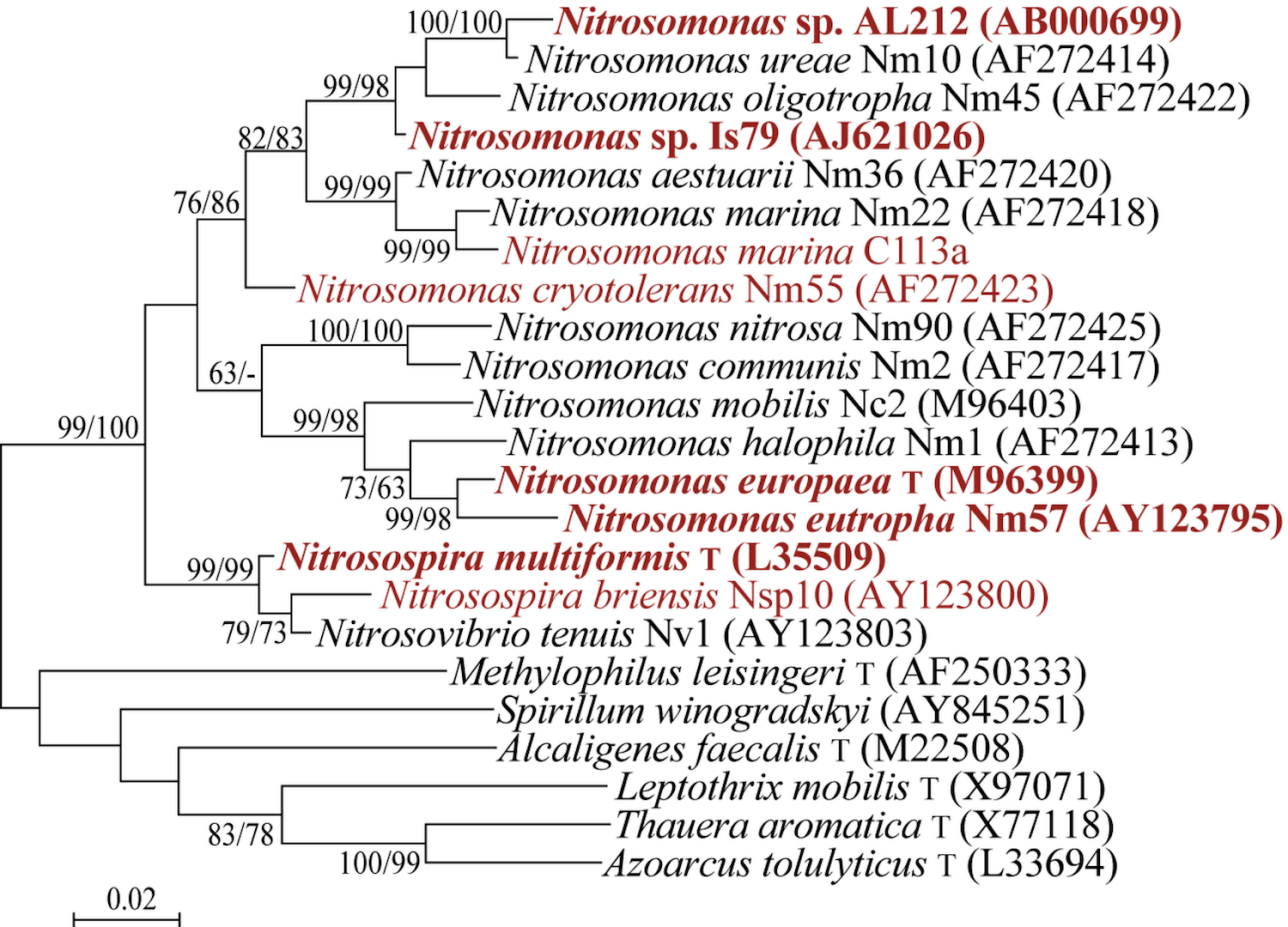
Phylogenetic tree showing the position of *Nitrosomonas sp.* Is79 relative to the other described strains within the family. *Nitrosomonas sp.* AL212 is not a formally described strain, but was included because the whole genome of this strain became recently available [12]. The tree was constructed from 1,272 aligned characters of the 16S rRNA gene sequence under the maximum likelihood criterion and rooted in accordance with a current taxonomy using the software package MEGA [35]. Numbers adjacent to the branches are support values from 1,000 ML bootstrap replicates (left) and from 1,000 maximum parsimony bootstrap replicates (right) if larger than 60% [35]. Strains with whole genome sequencing projects registered in GOLD [36] are shown in red and the published in red-bold: *Nitrosomonas europaea* (AL954747), *Nitrosomonas eutropha* (CP000450), *Nitrosospira multiformis* (CP000103), *Nitrosomonas sp.* AL212 (CP002552) and *Nitrosomonas sp.* Is79 (CP02876).

Strain Is79 was isolated into pure culture by A. Bollmann in 2001 and maintained in liquid stock cultures since then, being transferred to fresh medium approximately once per month. The strain has not been deposited in a culture collection, but can be obtained from A.B. upon request. Based on 16S rRNA gene sequences, the strains most closely related to *Nitrosomonas sp.* Is79 are *Nitrosomonas oligotropha* Nm10 with 97.8% sequence identity and *Nitrosomonas ureae* Nm45 with 97% sequence identity (Figure 1). The sequence of the single 16S rRNA gene copy in the genome of *Nitrosomonas sp.* Is79 differs by two nucleotides from the previously published 16S rRNA gene sequence (AJ621026), both of which are insertions into the whole genome sequence.

Growth studies show that *Nitrosomonas sp.* Is79 has a chemolithoautotrophic metabolism using ammonia as energy source producing nitrite. The strain is strictly aerobic and fixing carbon autotrophically from carbon dioxide via the Calvin cycle [37]. *Nitrosomonas sp.* Is79 is adapted to low ammonium concentrations and has been isolated after enrichment in continuous culture under ammonium-limited conditions [1]. Further experiments showed that *Nitrosomonas sp.* Is79 was able to grow and outcompete *Nitrosomonas europaea* under ammonium-limited conditions [38] and has K_s_ and K_m_ values for ammonium lower than other ammonia-oxidizing bacteria [Bollmann unpublished].

## Genome sequencing and annotation

### Genome project history

The organism was selected for sequencing as part of DOE-JGI program CSP 2006 because it is adapted to growth at low ammonium concentrations. The genome sequence is deposited in the Genome OnLine Database [36] and the complete genome is deposited in GenBank. Sequencing, finishing and annotation were performed by DOE-Joint Genome Institute (JGI). A summary of the project information is shown in Table 2.

**Table 2:** Genome sequencing project information

**MIGS ID**	**Property**	**Term**
MIGS-31	Finishing quality	Finished
MIGS-28	Libraries used	Three 454 pyrosequence libraries, standard and two paired end (9 and 7 kb average insert size) and one Illumina library
MIGS-29	Sequencing platforms	454 Titanium, Illumina
MIGS-31.2	Sequencing coverage	454 Titanium: 36.6 × and Illumina: 910.8 x
MIGS-30	Assemblers	Newbler version 2.3; VELVET version 1.0.13
MIGS-32	Gene calling method	Prodigal 1.4, GenePRIMP
	INSDC ID	CP002876
	GenBank Date of Release	July 05, 2010
	GOLD ID	Gc01870
	NCBI project ID	52837
	Database: IMG	2505679045
MIGS-13	Source material identifier	*Nitrosomonas sp.* Is79
	Project relevance	Environmental strain, nitrogen cycle

### Growth conditions and DNA isolation

The strain *Nitrosomonas sp.* Is79 was grown in mineral salts medium with 5mM ammonium at 27°C until all ammonium was consumed [39]. DNA was isolated using the protocol recommended by JGI (Bacterial genomic DNA isolation using CTAB). Size and quality of the bulk DNA was determined according to DOE-JGI guidelines. The size of the gDNA was larger than 23 kbp as determined by agarose gel electrophoresis.

### Genome sequencing and assembly

The draft genome of *Nitrosomonas sp.* Is79 was generated at the DOE Joint Genome Institute (JGI) using a combination of Illumina [40] and 454 technologies [41]. For the genome, we constructed and sequenced an Illumina GAii shotgun library which generated 46,913,976 reads totaling 3,565.5 Mb, a 454 Titanium standard library which generated 252,425 reads and 2 paired end 454 libraries with an average insert size of 7 kb, and 9 kb which generated 401,484 reads totaling 173.6 Mb of 454 data. All general aspects of library construction and sequencing performed at the JGI can be found at the JGI website [42]. The initial draft assembly contained 250 contigs in 3 scaffolds. The 454 Titanium standard data and the 454 paired end data were assembled together with Newbler, version 2.3-Prerelease-6/30/2009. The Newbler consensus sequences were computationally shredded into 2 kb overlapping fake reads (shreds). Illumina sequencing data were assembled with VELVET, version 1.0.13 [43] and the consensus sequence were computationally shredded into 1.5kb overlapping fake reads (shreds). We integrated the 454 Newbler consensus shreds, the Illumina VELVET consensus shreds and the read pairs in the 454 paired end library using parallel phrap, version SPS – 4.24 (High Performance Software, LLC). The software Consed [44-46] was used in the following finishing process. Illumina data were used to correct potential base errors and increase the consensus quality using the software Polisher developed at JGI [Lapidus, unpublished]. Possible mis-assemblies were corrected using gapResolution [Han, unpublished], Dupfinisher [47] or sequencing cloned bridging PCR fragments with subcloning. Gaps between contigs were closed by editing in Consed, by PCR and by Bubble PCR [Cheng, unpublished] primer walks. A total of 667 additional reactions were necessary to close gaps and to raise the quality of the finished sequence. The total size of the genome is 3,783,444 bp and the final assembly is based on 138.9 Mb of 454 draft data, which provide an average 36.6 coverage of the genome and 3,461Mb of Illumina draft data, which provide average 910.8× coverage of the genome.

### Genome annotation

Genes were identified using Prodigal [48] as part of the Oak Ridge National Laboratory genome annotation pipeline, followed by a round of manual curation using the JGI GenePRIMP pipeline [49]. The predicted CDSs were translated and used to search the National Center for Biotechnology Information (NCBI) nonredundant database, UniProt, TIGRFam, Pfam, PRIAM, KEGG, COG, and InterPro databases These data sources were combined to assert a product description for each predicted protein. Non-coding genes and miscellaneous features were predicted using tRNAscan-SE [50], RNAmmer [51], Rfam [52], TMHMM [53], and signal P [54].

## Genome properties

**Figure 2:**
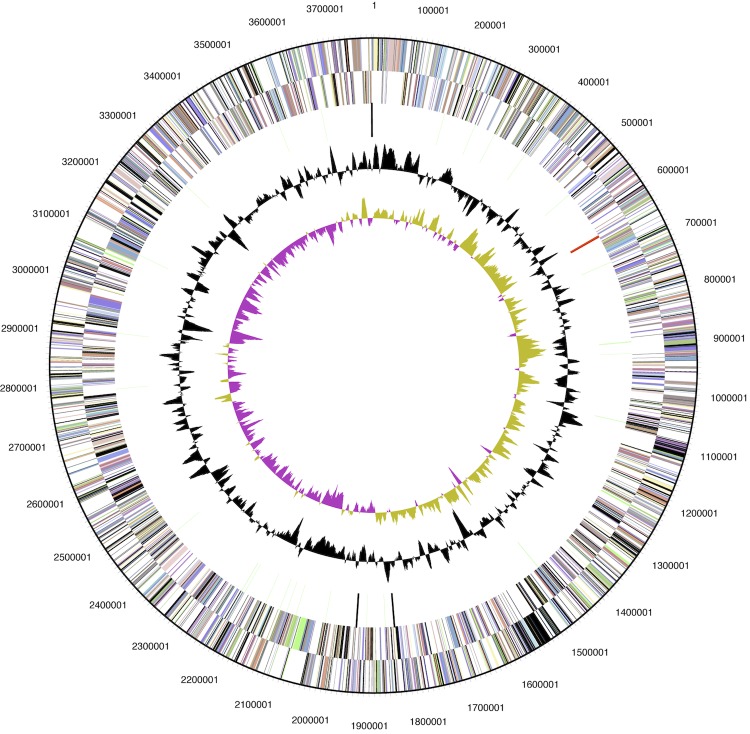
Graphical map of the genome. From the outside to the center: Genes on forward strand and Genes on reverse strand (color by COG categories), RNA genes (tRNAs green, rRNAs red, other RNAs black), GC content, GC skew.

The genome consists of a 3,783,444-bp long chromosome; the largest of all sequenced and published betaproteobacterial ammonia oxidizers [9-12]. The genome has a GC content of 45.4%. The genome contained 3,597 predicted genes of which 3,553 were protein-coding genes, 44 RNAs, and 181 pseudogenes (Figure 2). The majority of the protein-coding genes (63.64%) were assigned a putative function while the remaining ones were annotated as hypothetical proteins (Table 3). The distribution of genes into COGs functional categories is presented in Table 4.

**Table 3:** Genome statistics

**Attribute**	**Value**	**% of Total**
Genome size (bp)	3,783,444	100.00%
DNA coding region (bp)	3,166,256	83.69%
DNA G+C content (bp)	1,719,313	45.44%
Number of replicons	1	
Extrachromosomal elements	0	
Total genes	3,597	100.00%
RNA genes	44	1.22%
rRNA operons	1	
Protein-coding genes	3,553	98.78%
Pseudo genes	181	5.03%
Genes with function prediction	2,289	63.64%
Genes with paralog clusters	1,591	44.23%
Genes assigned to COGs	2,383	66.25%
Genes assigned Pfam domains	2,554	71.00%
Genes with signal peptides	1,130	31.42%
Genes with transmembrane helices	860	23.91%
CRISPR repeats	0	

**Table 4:** Number of genes associated with the general COG functional categories

**Code**	**Value**	**%age**	**Description**
J	167	6.34	Translation, ribosomal structure and biogenesis
A	7	0.27	RNA processing and modification
K	128	4.86	Transcription
L	270	10.24	Replication, recombination and repair
B	1	0.04	Chromatin structure and dynamics
D	39	1.48	Cell cycle control, cell division, chromosome partitioning
Y	0	0.00	Nuclear structure
V	49	1.86	Defense mechanisms
T	211	8.00	Signal transduction mechanisms
M	139	5.27	Cell wall/membrane/envelope biogenesis
N	94	3.57	Cell motility
Z	0	0.00	Cytoskeleton
W	0	0.00	Extracellular structures
U	87	3.30	Intracellular trafficking, secretion, and vesicular transport
O	132	5.01	Posttranslational modification, protein turnover, chaperones
C	146	5.54	Energy production and conversion
G	93	3.53	Carbohydrate transport and metabolism
E	152	5.77	Amino acid transport and metabolism
F	56	2.12	Nucleotide transport and metabolism
H	115	4.36	Coenzyme transport and metabolism
I	79	3.00	Lipid transport and metabolism
P	122	4.63	Inorganic ion transport and metabolism
Q	57	2.16	Secondary metabolites biosynthesis, transport and catabolism
R	259	9.83	General function prediction only
S	233	8.84	Function unknown
-	1214	33.75	Not in COG’s

## Insights from the genome sequence

### Ammonia monooxygenase

**Figure 3:**
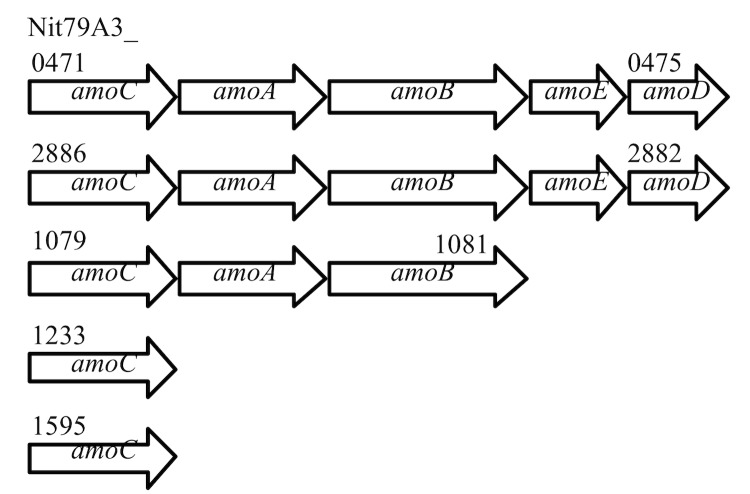
Organization of the *amo* gene clusters in the genome of *Nitrosomonas sp.* Is79.

The ammonia monooxygenase encodes the first enzyme in the oxidation of ammonia to nitrite via hydroxylamine [37]. Three *amoCAB* operons can be detected in the genome of *Nitrosomonas sp.* Is79 (Figure 3) and downstream of two of these operons the hypothetical genes (*amoE* and *amoD* [55]) were identified. The genome of *Nitrosomonas sp.* Is79 contains two single copies of the *amoC* gene. The copper resistance genes, *copC* and *copD* were not detected downstream of any of the *amoCAB* operons as it was identified in all other described betaproteobacterial ammonia oxidizers [9-12,56].

## Hydroxylamine oxidoreductase

The hydroxylamine oxidoreductase (HAO) is the second enzyme in ammonia oxidation, catalyzing the oxidation of hydroxylamine to nitrite [37]. As all other betaproteobacterial ammonia oxidizers, *Nitrosomonas sp.* Is79 encodes three syntenous *hao* operons consisting of the genes *haoA* encoding the octaheme cytochrome *c* protein subunit that forms the functional HAO complex, *haoB* encoding an uncharacterized gene product, *cycA* encoding cytochrome c554, and *cycB* encoding the quinone reductase, cytochrome c_M_552.

### Nitrogen oxide metabolism

A copper-containing nitrite reductase (*nirK*) was detected in the genome of *Nitrosomonas sp.* Is79. As detected in *N. multiformis* and *Nitrosomonas sp.* AL212, the *nirK* gene exists as a singleton in the genome, which is in contrast to its position in the genomes of *N. europaea* and *N. eutropha* where *nirK* is a member of a conserved multigene cluster (Table 5) [9-12,56].

**Table 5:** Presence and absence of genes involved in nitrogen oxide metabolism based on [9-12]

**Genes**		NE*	Neut*	Nmul*	NAl212*	Nit79A3*
Nitrite reductase	*nirK*	+	+	+	+	2335
Nitrogen sensitive transcriptional regulator	*nsrR*	+	+	-	-	-
Nitric oxide reductase	*norCBQD*	+	+	+	+	-
Nitric oxide detoxification						
Heme-copper nitric oxide reductase	*norSY-senC-orf1*	+	+	+	-	-
Cytochrome c′-beta	*cytS*	+	+	+	+	0363
Cytochrome P460	*cytL*	+	+	-	+	1628
Nitrosocyanin	*ncyA*	+	+	+	+	-

* NE: *N.europaea*; Neut: *N.eutropha*; Nmul: *N.multiformis*; NAL212: *Nitrosomonas sp.* AL212; Nit79A3: *Nitrosomonas sp.* Is79

(+ presence of the gene in the genome; - absence of the gene from the genome; numbers present the position of the genes in the genome of *Nitrosomonas sp.* Is79)

The nitrite or nitric oxide responsive transcription factor *nsrR* [57] is missing in the genome of *Nitrosomonas sp.* Is79, indicating that *Nitrosomonas sp.* Is79 might have different response mechanisms to nitrite and nitric oxide than *N. europaea* or *N. eutropha* (Table 5) [9,10].

Genes encoding enzymes for nitric oxide reduction to nitrous oxide (*norCBQD*) were found in all betaproteobacterial ammonia oxidizers except again in *Nitrosomonas sp.* Is79. Genes found in the genomes of most chemolithotrophic ammonia oxidizers encoding additional inventory implicated in nitric oxide detoxification to prevent nitrosative stress (cytochrome P460, cytochrome *c′*-beta [58];) (Table 5) were also identified in the genome *Nitrosomonas sp.* Is79; however, the genes encoding sNOR were absent. Based on these results it is very likely that *Nitrosomonas sp.* Is79 can avoid nitrosative stress caused by nitric oxide [59]. In addition, it is likely that *Nitrosomonas sp.* Is79 cannot reduce nitric oxide to nitrous oxide via nitrifier denitrification [60], but may use an alternate pathway as demonstrated for the nitrifying methanotroph, *Methylococcus capsulatus* strain Bath [61,62].

Finally and in contrast to all other betaproteobacterial ammonia oxidizers, the gene encoding the red copper protein nitrosocyanin [63] was not identified in the genome of *Nitrosomonas sp.* Is79. It is currently unclear what implications the absence of this gene may have on the metabolism of *Nitrosomonas sp.* Is79, because the function of the protein itself is still elusive.

### Ammonia transporter

The gene encoding an ammonia transporter (amtB type) was detected in the genome of *Nitrosomonas sp.* Is79. Ammonia transporters are needed for the acquisition of ammonia/ammonium for assimilation. The function of these genes in ammonia oxidizers that are adapted to low ammonium concentrations is of particular interest also because the process of nitrogen assimilation competes directly with the bacterium’s need for ammonia to sustain catabolism or the generation of energy.

### Urease

The enzyme urease is responsible for hydrolyzing urea to yield ammonium and carbon dioxide, thereby increasing the substrates for N and C assimilation in the cytoplasm. While the genome of *Nitrosomonas sp.* Is79 lacks the gene cluster encoding urea hydrolase (*ureABCDEFJ*) [64]; the genes encoding biotin-containing urea carboxylase and the putative allophanate hydrolase were detected. It has been suggested that the products of these genes convert urea to ammonium and carbon dioxide while consuming metabolic energy (ATP). Incubation of *Nitrosomonas sp.* Is79 in the presence of urea did not result in the production of nitrite [Sedlacek and Bollmann, unpublished] indicating that urea was not degraded, and that expression of these genes might be regulated through a network controlled by the energy status of the cell.

### Hydrogenase

The genome of *Nitrosomonas* Is79 contained most of the putative [NiFe] hydrogenase-encoding genes found in the genome of *N. multiformis* [11]. However, one of the hypothetical proteins is missing, and the genes are scattered over the genome instead of being members of single gene cluster as in *N. multiformis* [11].

### Carbon dioxide fixation

**Figure 4:**
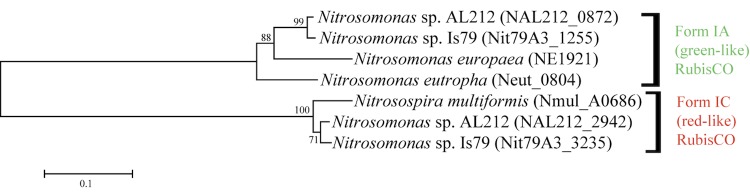
Phylogenetic tree of betaproteobacterial ammonia oxidizers inferred using the Maximum Likelihood criterion using the software package MEGA [35] based on the protein sequence of the large subunit of the RuBisCO (*cbbL*). The alignment was inferred by ClustalW software [35]. Numbers adjacent to the branches are support values from 1,000 ML bootstrap replicates if higher than 60% [35].

As observed in all ammonia-oxidizing bacteria, *Nitrosomonas sp.* Is79 fixes carbon dioxide via the Calvin cycle involving the main enzyme RuBisCO (Ribulose-1,5-bisphosphate carboxylase oxygenase). The genomes of *Nitrosomonas sp.* Is79 and *Nitrosomonas sp.* AL212 [12] encoded two copies of the RuBisCO operon (Figure 4). One copy belongs to form IA (green-like) RuBisCO and is closely related to the RuBisCO in *N. europaea* and *N. eutropha*, while the other copy belongs to form IC (red-like) RuBisCO and is closely related to the enzyme in *N. multiformis* (Figure 4). The form A RuBisCO is not associated with the genes for the carboxysome as in *N. eutropha* [10]. The two RuBisCO copies differ in their kinetic properties. *Bacteria* with RuBisCO form IA have a higher affinity for carbon dioxide than organisms with RuBisCO form IC [65]. Therefore it is very likely that ammonia oxidizers with two different gene copies of the RuBisCO gene have a higher flexibility with respect to the carbon dioxide availability in the environment.

### Other genes of interest

When comparing the genome of *Nitrosomonas sp.* Is79 with the other available genomes of betaproteobacterial ammonia oxidizers, several genes and operons were detected that were missing in or unique to the investigated strain.

### Potassium transporters

The genomes of *N. europaea, N. eutropha*, *N. multiformis* and *Nitrosomonas sp.* AL212 encode the genes *phaABCDEFG* for a NADH driven potassium (cation) proton antiporter. While this potassium transporter was not detected in the genome of *Nitrosomonas sp.* Is79, the genes for another high affinity ATP driven potassium transporter (*kdpABC*) were found. These three genes encoding the potassium transporter ATPase (Nit79A3_1970-1972) were upstream of an osmosensitive signal transduction histidine kinase (*kdpD*) and a two component transcriptional regulator (*kdpE*). The kdp operon encodes an inducible high affinity potassium transport system that will be expressed under potassium deficiency [66,67]. *Nitrosomonas sp.* Is79 is known to be adapted to low nutrient concentrations and oligotrophic conditions. The presence of this high affinity transport system could be an adaptation to low ion strength environments.

### Iron transport

The genome of *N. europaea* was characterized by a high number of different kinds of iron transporters [9] and all other genomes including *Nitrosomonas sp.* Is79 contained high affinity iron transporters. In addition a low affinity iron permease was detected in the genome of *Nitrosomonas sp.* Is79 (Nit79A3_3148). This enzyme has been characterized well in *Saccharomyces cerevisiae* and is involved in transport of iron, copper and zinc [68]. These authors discuss the possibility that the high affinity transport systems are active under limiting conditions, while the iron permease becomes active under non-limiting conditions. The enzyme might have the same function in *Nitrosomonas sp.* Is79.

## Conclusion

The genome of *Nitrosomonas sp.* Is79 is the largest betaproteobacterial ammonia oxidizer genome sequenced to date. The genome shows differences in gene content when compared to other betaproteobacterial ammonia oxidizers, some of which might have importance for the adaptation to low environmental ammonia concentrations. We believe that the study of this inventory – missing or unique - will help to elucidate the adaptation of ammonia oxidizers to oligotrophic environments.
